# Spanish experience of an extensively hydrolyzed formula with lactose and human milk oligosaccharides in cow’s milk allergy

**DOI:** 10.1186/s12875-026-03481-5

**Published:** 2026-07-23

**Authors:** Cristóbal Coronel-Rodríguez, Marta Castell Miñana, Miquel Layola Brias, participants from ELEVA study, Ainhoa Izquierdo, Alba Alcaraz, Alba Oviedo, Amaia Iglesias, Amalio Fernández, Ana M. Nocea, Ana Martín, Antonio J. Castellanos, Borja Navas, Carme Valls, Carmen Martín, Carmen Torres, Cristina M. Fresno, Daniel Gómez, David González, Elena Aymerich, Elena M. García, Francisco J. Sola, Henry A. Luján, Javier Perona, Jorge López, Jose L. Molina, Julia Fujkova, Maria A. Carreño, María del Mar Roncero, María D. Sebastia, María I. Irles, María L. Arroba, Marianna A. Di Campli, Marta Sifre, Miguel A. Monroy, Olga González, Ainhoa Muguruza, Alba de la Mata, Alicia Lizondo, Almudena Navarro, Álvaro Mula, Amalia Cruz, Amparo S. Romero, Ana Asensio, Ana I. Jiménez, Ana I. Rodríguez, Ana J. Llanes, Ana M. Monago, Ana M. González, Ana M. Hervás, Ana M. Sánchez-Cañete, Ángel Mir, Angela Claver, Anna Pizà, Antonia Díez, Antonio Almagro, Antonio de Andrés, Antonio García, Antonio Giráldez, Antonio Rosell, Bárbara Corvillo, Beatriz Lorenzo, Carlos Núñez, Carmen Hernandez, Carolina Blanco, Carolina I. Boccardo, Carolina Valle, Cintia C. Ago, Claudia Vásquez, Conchi Gonzalo, Cristina M. López, Cristina Ortola, Cristina Royo, Cristina Sevilla, Cristóbal Coronel, Cynthia Crespo, Diana Torres, Eduardo Haro, Elia Zoido, Elisa Gutiérrez, Enric Giménez, Enrique La Orden, Enrique Llerena, Erika Otazu, Esther Apolinar, Eva Carvajal, Eva Palaci, Fátima Diaz, Fátima M. Violadé, Francisco Fernández, Francisco J. Jerez, Francisco J. Recio, Francisco J. Ponce, Gemma Colomé, Georgina Catasús, Gisela Roca, Gloria Giner, Gonzalo Botija, Gonzalo Galicia, Guillermo Longo, Ignacio Domingo, Ignacio Güemes, Ilse Medrano, Inés Cervera, Irene García, Irene M. Satorre, Janice M. Quiroz, Javier Artero, Javier Villalba, Jesús González, Jesús Rodríguez, Joaquín Roig, Jorge Martínez, José J. Ruiz, Juan C. Juliá, Juan I. Manrique, Juan J. Roeschlin, Juan P. Ferrer, Kilian Arias, Laura Calvo, Laura Orsola, Laura Quinzá, Laura Viñas, Leandro Picó, Leticia Postigo, Lexuri Zugadi, Lidia Aulet, Lorena Hidalgo, Lorena Rodríguez, Lucia Barahona, María T. Joval, María A. Tormos, Mara Cerqueiro, María A. Sarría, Maria A. Mate, María del Mar Corral, Maria D. Saavedra, Maria E. Jaime, Maria J. Serrano, María Martínez, Maria T. Alcayde, Maria T. Guerra, Maria Valls, Mariel Navarro, Marina García, Marina Rodríguez, Marta Espinet, Marta Garín, Marta Muñoz, Marta Pozuelo, Marta Sarría, Miguel Gallardo, Mireia Corominas, Mónica García, Montserrat Hernández, Neelam M. Dadlani, Neyda A. Landaeta, Nieves García, Nieves Torres, Noelia Ruiz, Nuria Barros, Nuria Doñate, Nuria Puente, Nuria Roig, Nuria Sanchis, Octavio Berbel, Olga K. Maco, Oliva Piera, Pablo O. Goicolea, Paloma Cañete, Patricia Villegas, Paula Ribó, Paula Soriano, Rebeca Corral, Rocio Collado, Rocío Mendívil, Rocío Ramírez, Sandra Aycart, Sandra Mayola, Sandra Rodríguez, Sergio Negre, Silvia Monteagudo, Silvia Pin, Souheil El-Solh, Susana Domínguez, Yohanka Rabelo, Yusty M. Herazo, Zoraima Martínez, Paula Gacimartín, Salvador Ariza, Silvia Marqués, Susana Pardo, Susana Villanueva, Víctor Fernández, Violeta Campos, Lusmey Fernández, Feliciano Rubio

**Affiliations:** 1 Pediatrics Service, Amante Laffón Healthcare Centre, Seville, Spain; 2 Pediatrics Service, Campanar Healthcare Center, Valencia, Spain; 3 Medical Affairs Manager, Nestlé Health Science Spain, Barcelona, Spain; 4 Nestlé Health Science Spain, Carrer de Clara Campoamor, 2. 08950 - Esplugues de Llobregat, Barcelona, Spain

**Keywords:** Cow's milk protein allergy, Extensively hydrolyzed formula, Lactose, Human milk oligosaccharides, Primary care, Spain, Ecological

## Abstract

**Background:**

Despite the evidence supporting the efficacy of extensively hydrolyzed formulas (eHF) and human milk oligosaccharides (HMO) for the management of cow’s milk protein allergy (CMPA), data on their use in clinical practice remain limited. The objective of the study was to describe the current landscape of CMPA management in Spanish primary care consultations, with particular emphasis on eHFs and novel immunomodulatory components (purified lactose or HMO).

**Methods:**

ELEVA was a multicenter, descriptive, ecological study involving 191 pediatricians with experience on CMPA management. Aggregated data derived from their experience on CMPA management.

**Results:**

The eHFs were the most commonly prescribed first-line option for mild and moderate CMPA (78.5% and 95.8%, respectively), while elemental formulas were mainly used in severe cases (61.8%). Infants who received an eHF supplemented with purified lactose and two HMO improved their digestive symptoms (77.4%), showed a reduction of crying and/or irritability symptoms (63.1%), normalization of stool consistency (51.6%), avoidance the rejection/difficulty for eating (48.2%), or a reduction of the number of stools (44.9%).

**Conclusions:**

The ELEVA study offers detailed insights into how pediatricians in Spain manage CMPA in clinical practice. It also highlights the integration of innovative tools, such as the CoMiSS™ awareness tool for CMPA diagnosis, and novel treatment components like purified lactose and HMO. The use of an eHF supplemented with purified lactose and HMO appears effective in alleviating digestive issues and irritability symptoms. Additionally, it may influence the incidence of infections, use of medication, and number of visits to the healthcare center.

**Supplementary Information:**

The online version contains supplementary material available at https://doi.org/10.1186/s12875-026-03481-5.

## Introduction

Cow’s milk protein allergy (CMPA) is the most common food allergy in infants and young children, affecting 2–3% of children in Spain [1]. CMPA is the result of an abnormal immune response to cow’s milk proteins, and that can be mediated through immunoglobulin E (IgE), non-IgE pathways, or a combination of both [2, 3]. In 60% of cases, symptoms appear after the initial ingestion of milk, and rarely, after a week [3]. CMPA symptoms vary widely, affecting the digestive system (50–60% of cases), skin (50–60%), or the respiratory tract (20–30%) [2, 3]. Anaphylaxis is the most severe reaction, occurring in approximately 0.8–9.0% of cases. The safest strategy for managing CMPA is the elimination diet [4]. For exclusively breastfed infants who experience chronic symptoms, it is recommended that mothers avoid any dairy products. For non-exclusively breastfed infants, international scientific societies, such as the European Society of Pediatric Gastroenterology, Hepatology, and Nutrition (ESPGHAN), recommend an extensively hydrolyzed formula (eHF) with proven efficacy in appropriately designed clinical studies as the first option, or an amino acid based formula (AAF) based on free L-amino acids for cases of severe allergic reactions [4]. Guidelines also indicate that eHFs containing purified lactose are considered safe and effective for the treatment of CMPA, while additionally offering a more palatable taste.

On the other hand, human milk oligosaccharides (HMO) are approximately 200 molecules synthesized by the mammary gland and naturally present in human milk [5, 6]. They play a fundamental role in the establishment of the intestinal microbiota, protection against infections, and the development of the immune system, as well as intellectual development during the first years of life. Diverse studies have demonstrated that formula supplementation with HMO provides beneficial effects on immunological, intestinal, bone, and cognitive development in healthy infants, as well as in cases of allergies, premature, or infants with CMPA [7–11].

Despite the evidence supporting the efficacy of eHFs and HMO for the management of CMPA, data on their use in routine clinical practice remain limited [12, 13]. The objective of the study was to describe the current landscape of CMPA management in Spanish primary care consultations, with particular emphasis on the use of eHFs added with purified lactose or HMO.

## Methods

### Study design

ELEVA was a multicenter, descriptive, ecological study involving pediatricians with experience on CMPA management from primary care healthcare centers of Spain. The data source was the pediatricians’ experience on the management of CMPA, collected through a questionnaire specifically designed for this purpose. The study focused on analyzing the population as a whole (aggregated data). Therefore, none of the data were obtained from medical records or individual infant information. The aggregated data derived from the pediatrician’s last five infant with CMPA to whom pediatricians prescribed a hypoallergenic whey based eHF supplemented with purified lactose and two HMO, i.e. 2′fucosyl-lactose (2’-FL) and lacto-N-neotetraose (LNnT).

### Specifically designed questionnaire

A coordinating group, led by the authors CC-R, MCM, and MLB, developed the questionnaire based on their clinical experience. The questionnaire consisted of 31 items, covering information about the participating pediatrician (three items), the diagnosis and nutritional management of CMPA (nine items), the sociodemographic and clinical characteristics of infant with CMPA (five items), and the use of purified lactose and HMO for the nutritional management of the last five infants with CMPA treated by them (14 items). The questionnaire (translated to English) is shown in the Appendix S1. Pediatricians completed the questionnaire via an online platform. Participants responded regarding the diagnosis and management of CMPA based on their overall experience with infants. When referring to the use an eHF supplemented with purified lactose and two HMO, participants based their responses on their experience with the last five infants to whom they had prescribed the formula.

### Ethical considerations

All participants agreed to participate in the study by signing an informed consent form. Ethical approval for the study was granted by the Ethics Committee of the Hospital Clínico San Carlos (Madrid, Spain). Procedures were conducted in accordance with national legislation and the Declaration of Helsinki.

### Statistical analysis

Categorical variables are described using absolute and relative frequencies. All descriptive procedures were analyzed by using the Statistical Package for the Social Sciences (SPSS) version 22.0 (SPSS Inc., Chicago, IL, USA).

## Results

### Participating pediatricians

Between September 2023 to January 2024 a total of 191 pediatricians from primary care healthcare centers of 12 Autonomous Communities of Spain participated in the study (Appendix S2). Pediatricians had more than ten years of experience in CMPA (66.5%).

### Overall diagnosis of CMPA in infants

Among participants, 79.6% stated that CMPA diagnosis was mainly established based on clinical symptoms. Of pediatricians, 45.5% used a tool for detecting and monitoring potential CMPA symptoms, particularly the Cow's Milk-related Symptom Score (CoMiSS™; 52.9%; Appendix S3).

### Overall prescribing practices and decision-making for infants with CMPA

The eHFs were the most commonly prescribed first-line option for mild and moderate CMPA (stated by 78.5% of participants and 95.8%, respectively), while elemental formulas were mainly used in severe cases (61.8%; Fig. 1). The main factors guiding formula selection were the degree of hydrolyzation (82.6%), the strength of the supporting scientific evidence (58.4%), organoleptic properties (52.1%), and protein type (47.4%; Appendix S4). The main reason for the prescription of an eHF supplemented with HMO was its capacity to promote the growth of beneficial bacteria and, consequently, to support immune system development (84.8%; Appendix S5). Regarding an eHF supplemented with purified lactose, main reasons for its prescription included the improved palatability and reduced risk of rejection (34.0%), being a fundamental component of human milk (28.8%), and its contribution to facilitating calcium absorption (15.2%; Appendix S6).

**Fig. 1:**
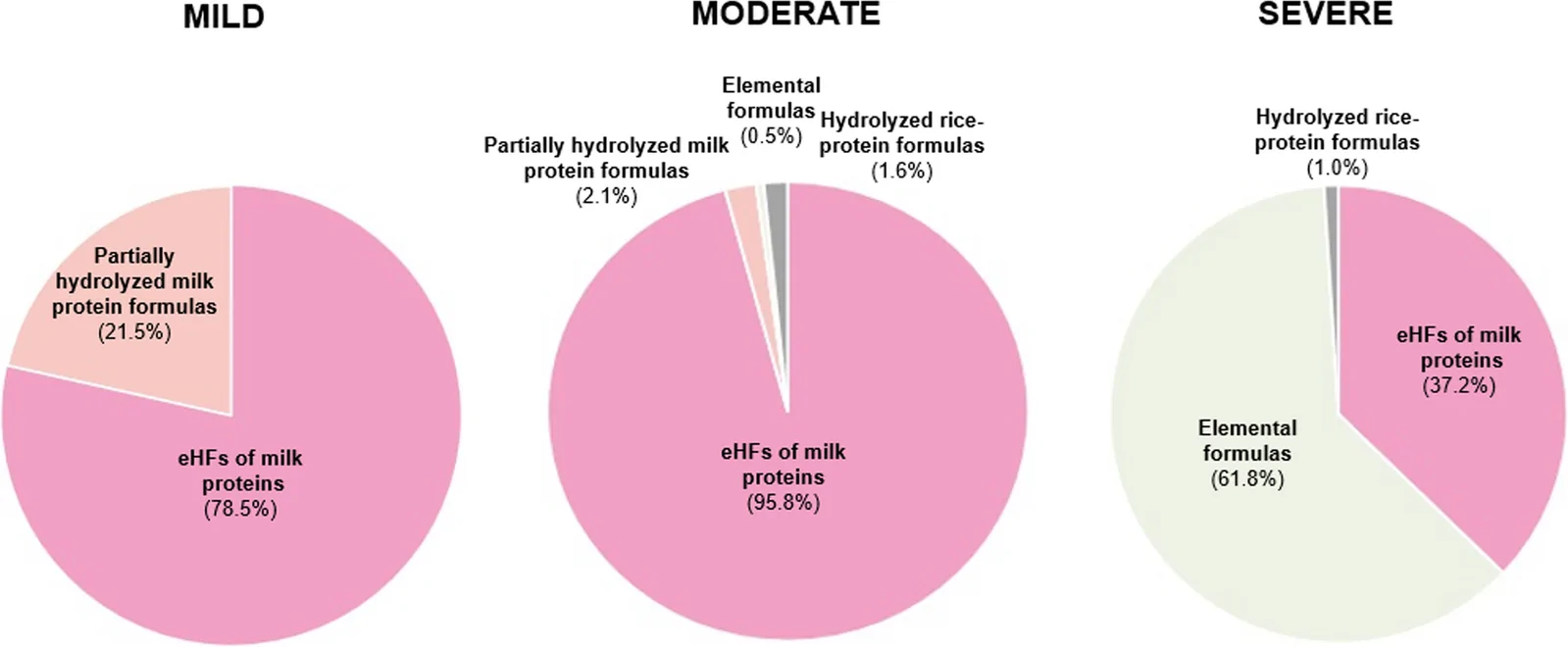
Most frequent first-line formulas prescribed in infants with CMPA regarding its severity. CMPA, cow’s milk protein allergy

### Usage of an eHF supplemented with purified lactose and two HMO

Of pediatricians, 81.8% stated that infants who received an eHF supplemented with purified lactose and two HMO (2’-FL and LNnT) were predominantly aged between 0 and 6 months, 75.5% that showed a non-IgE-mediated CMPA, and 62.2% and 33.5% that presented with mild and moderate severity (Appendix S7). They improved their digestive symptoms (stated by 77.4% of participants), showed a reduction of crying and/or irritability symptoms (63.1%), normalization of stool consistency (51.6%), avoidance the rejection/difficulty for eating (48.2%), or a reduction of the number of stools (44.9%; Fig. 2). Of pediatricians, 57.0% indicated that symptom improvement occurred within the first week after the administration of the formula (Fig. 3). Pediatricians reported that the formula was associated with a lower incidence of respiratory tract infections (50.7%), reduced use of medications such as antipyretics and antibiotics (35.9%), and a lower number of healthcare visits (40.6%) compared with an extensively hydrolyzed formula (eHF) lacking purified lactose or HMO (Fig. 4). The formula was prescribed mainly due to its role in immune system maturation (86.4%), symptom relief (83.8%), and its breast milk–like composition (80.1%; Appendix S8). The degree of satisfaction with the use of an eHF with purified lactose and HMO was mostly very satisfied (60.7%) or satisfied (37.7%; Appendix S9).

**Fig. 2:**
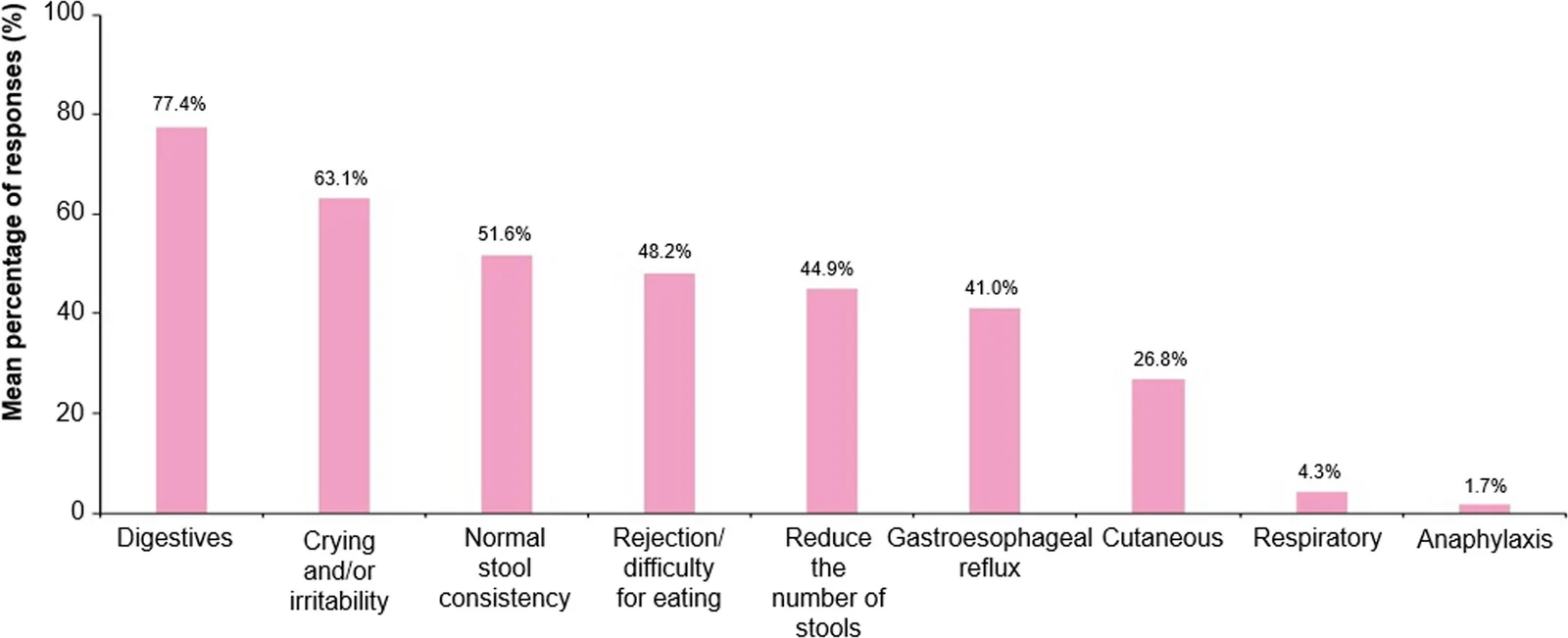
Mean percentage of infants showing improvement of CMPA symptoms. CMPA, cow’s milk protein allergy

**Fig. 3:**
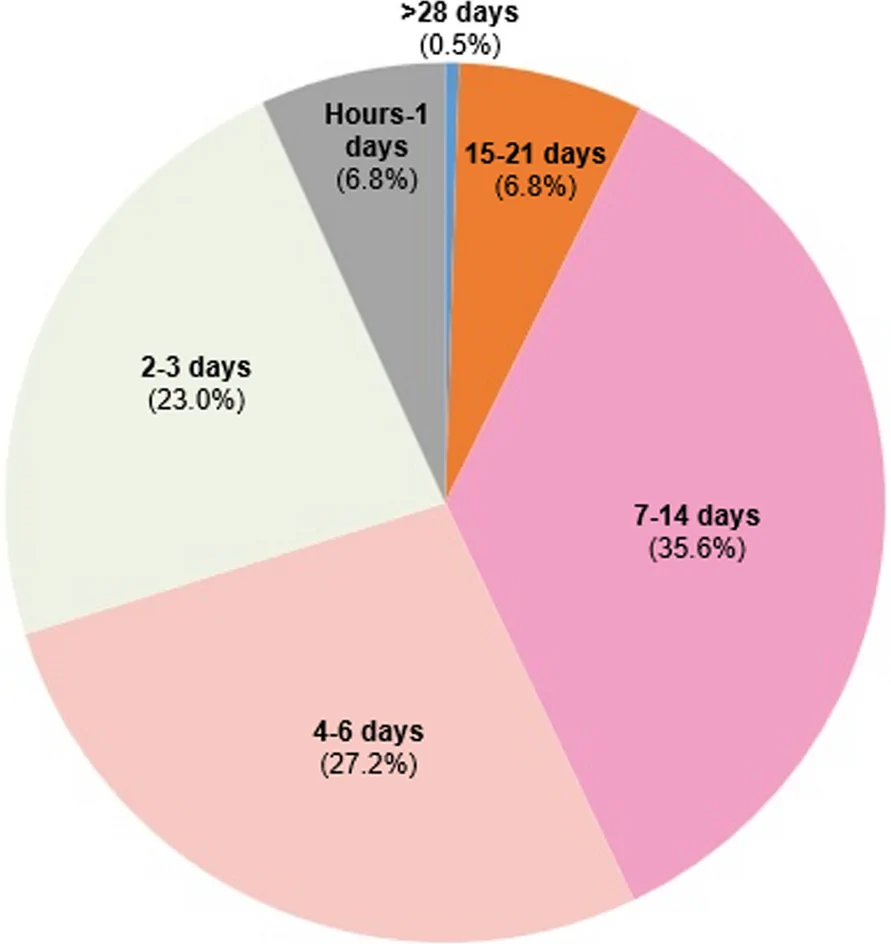
Time to improvement of symptoms after the administration of the formula

**Fig. 4:**
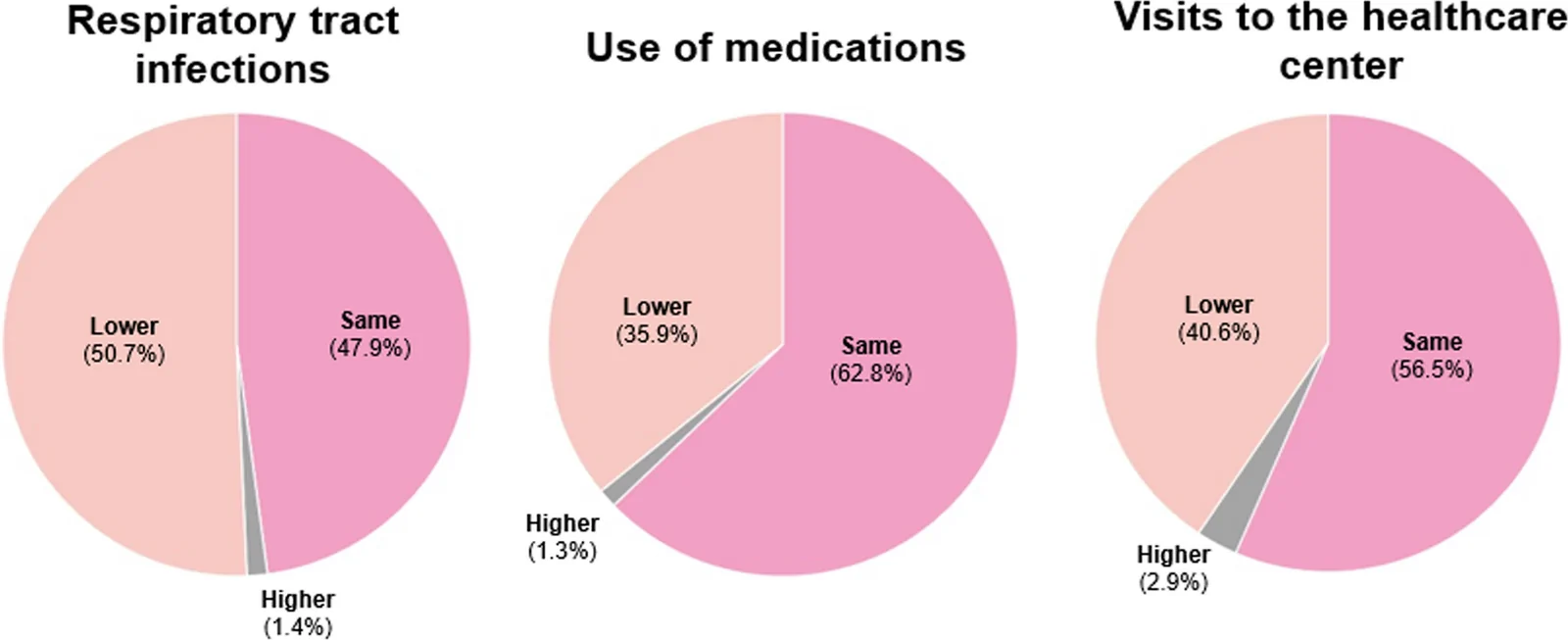
Opinion of the pediatricians on the use of eHF with lactose and HMOs versus another without purified lactose and HMOs regarding diverse variables. eHF, extensively hydrolyzed formula; HMOs, human milk oligosaccharides

## Discussion

Our study shows updated insights into the diagnosis, prescribing practices, and use of an eHF supplemented with purified lactose and two HMO for the management of CMPA in Spain. The diagnosis of CMPA was based on symptomatology presented in infants. CMPA manifestations are classified into mild, moderate, and severe categories [2–4]. Nevertheless, the non-specificity of symptoms involved in CMPA may result in misdiagnosis, which in turn may lead to growth impairment, increased stress, and greater healthcare costs [14]. In our study, approximately one in two pediatricians employed a tool for detecting and monitoring potential CMPA symptoms; and among them half of them used CoMiSS™. CoMiSS™ is a rapid, concise, and easy-to-use awareness tool based on symptoms (such as crying, gastrointestinal, dermatological, and respiratory symptoms) [15]. Diverse studies have demonstrated the utility of the tool for the diagnosis of CMPA [16]. Despite this, a multicenter survey conducted in Spain with 294 pediatricians revealed that only approximately one third of infants diagnosed with CMPA exhibited a CoMiSS™ of 12 points or higher at the time of evaluation, indicating inadequate use of the tool in clinical practice and a lack of knowledge among pediatricians [17]. In our study, pediatricians knowledge led them to prescribe an eHF for mild and moderate CMPA, and an elemental formula for severe cases. This observation is in agreement with clinical guidelines [4]. However, it is important to note that in our study, eHF was still used in 37.2% of severe CMPA cases, instead of the recommended use of AAF. In addition, 21.5% and 2.1% of patients with mild and moderate CMPA, respectively, received partially hydrolyzed formulas instead of the recommended eHF. Altogether, these findings highlight the need to strengthen education of HCPs for promoting the adherence to international guidelines. In our study, when choosing an eHF, pediatricians primarily considered the degree of hydrolyzation, the scientific evidence supporting the formula, organoleptic properties (such as palatability), and the type of protein. Main reasons reported by pediatricians for prescribing an eHF supplemented with HMOs included its potential to promote immune system maturation, its effectiveness in relieving clinical symptoms, and its similarity to the composition of human breast milk.

Our study also showed that the use of the eHF appeared to contribute to the reduction of digestive and irritability symptoms, while also decreasing the incidence of infections and the use of healthcare resource. This observations are in concordance with found in previous studies [7, 10, 11, 18]. For example, the randomized, controlled, double-blind, multicenter CINNAMON clinical trial determined whether supplementing an eHF with two HMO (2’-FL and LNnT) and reduced protein content (2.2 g/100 kcal) supported normal growth in infants with CMPA [10]. Investigators recruited 194 infants (aged 0 to 6 months) and followed them up for four months. Results indicated that weight gain in infants receiving the HMO-supplemented formula was non-inferior to that of the control group (eHF without HMO). No significant differences were observed in anthropometric measures between the groups [10]. Infants on the HMO formula experienced a significant 42% reduction in the monthly frequency of upper respiratory tract infections and a clinically relevant trend of 30 to 40% relative risk reduction in lower respiratory tract infections and gastro-intestinal infections.

Our present study is interesting because it represents the first one in depicting the clinical use of an eHF supplemented with purified lactose and HMO in Spain. However, it presents some limitations that should be taken into account. The main one derives from the subjective nature of the data, which were provided as aggregated data and based on pediatricians' clinical experience, without including information collected from medical records or using effectiveness outcomes. As a consequence, data might also be influenced by recall and subjective bias. Yet, ecological studies are a type of the observational designs in epidemiology, and are used as hypothesis generators due to their simplicity, speed, low cost, and relatively straightforward analysis [19]. For this reason, our results should be considered as an initial step in understanding the current management of CMPA, as well as the use of eHFs and purified lactose and HMO in primary care of Spain. Furthermore, the study was funded by Nestlé, and therefore the responses of pediatricians may have been subject to potential bias. Accordingly, findings should be interpreted with caution; however, our results are consistent with those reported in previous studies [4, 10].

## Conclusion

The ELEVA study offers detailed insights into how pediatricians in Spain manage CMPA in clinical practice. It also highlights the integration of innovative tools, such as the CoMiSS™ awareness tool for CMPA diagnosis, and novel treatment components like purified lactose and HMO. The use of an eHF supplemented with purified lactose and HMO appears effective in alleviating digestive issues and irritability symptoms. Additionally, it may influence the incidence of infections, the use of medication, and the number of visits to the healthcare center.

## Supplementary Information

Supplementary Material 1.

## Data Availability

The datasets used and/or analyzed during the current study are available from the corresponding author on reasonable request.

## Abbreviations

AAF: Amino acid based formula
CMPA: Cow’s milk protein allergy
eHF: Extensively hydrolyzed formula
ESPGHAN: European Society of Pediatric Gastroenterology, Hepatology, and Nutrition
HMO: Human milk oligosaccharides
IgE: Immunoglobulin E
SPSS: Statistical Package for the Social Sciences
