# Supplementary material for: Spanish experience of an extensively hydrolyzed formula with lactose and human milk oligosaccharides in cow’s milk allergy
Source: BMC Prim Care. 2026 Jul 23;27:379. doi: 10.1186/s12875-026-03481-5 (PMC13681903; doi:10.1186/s12875-026-03481-5)
Supplement: Supplementary file 1 [file 12875_2026_3481_MOESM1_ESM.docx]

# **APPENDIX S1**

# **Questionnaire**

Here you will find a questionnaire in which, first of all, you will have to fill in information related to you (General data of your professional profile), and, subsequently, your patients. In each of these sections you will find different fields to be filled in:

- Fields with “_____”: are open fields to write text or numbers.
- Fields with “o”: only one option can be selected.
- Fields with “□”: more than one option can be selected.

**BLOCK 1: GENERAL DATA OF YOUR PROFESSIONAL PROFILE**

1. Type of healthcare center where you attend pediatric patients with cow's milk protein allergy (CMPA):

- Public
- Private
- Both

***Note:*** *only one option can be selected.*

2. Region to which the healthcare center (where you attend pediatric patients with CMPA) belongs:

- Andalucía
- Aragón
- Canarias
- Cantabria
- Castilla-La Mancha
- Castilla y León
- Cataluña
- Ciudad Autónoma de Ceuta
- Ciudad Autónoma de Melilla
- Comunidad de Madrid
- Comunidad Foral de Navarra
- Comunidad Valenciana
- Extremadura
- Galicia
- Illes Balears
- La Rioja
- País Vasco
- Principado de Asturias
- Región de Murcia

***Note:*** *only one option can be selected.*

3. Number of years of experience you have managing CMPA:

- 1-5
- 6-10
- 11-15
- 16-20
- >20

***Note:*** *only one option can be selected.*

**BLOCK 2. DIAGNOSIS AND NUTRITIONAL TREATMENT OF CMPA**

To complete the following questions, please take into account your routine clinical practice on the diagnosis and nutritional treatment of CMPA. You must ensure that the data provided are as close as possible to what happened in your consultation.

4. On what do you mainly base the diagnosis of CMPA in a pediatric patient?

- On the symptomatology (compatible with CMPA)
- On the elimination diet
- On the oral food challenge test
- On the atopy patch test
- On the serum specific IgE test
- On the s*kin prick test*

***Note:*** *only one option can be selected*

5. Do you use any tool for the detection and evolution of potential CMPA symptoms?

- Yes. If so, please indicate which one: ___________
- No

***Note:*** *only one option can be selected.*

6. Would you consider necessary the implementation of tools for the detection and evolution of potential CMPA symptoms?

- Yes, but only to support the detection of CMPA
- Yes, but only for the evolution of symptoms
- Yes, for all cases
- No

***Note:*** *only one option can be selected.*

7. What is the MOST FREQUENT type of formula do you prescribe as first-line for CMPA, depending on its severity?

| Mild | - Partially hydrolyzed milk protein formulas - Extensively hydrolyzed formulas (eHFs) of milk proteins - Hydrolyzed soy formulas - Hydrolyzed rice-protein formulas - Elemental formulas (based on free amino acids) - Other   ***Note:*** *only one option can be selected.* |
| --- | --- |
| Moderate | - Partially hydrolyzed milk protein formulas - eHFs of milk proteins - Hydrolyzed soy formulas - Hydrolyzed rice-protein formulas - Elemental formulas (based on free amino acids) - Other   ***Note:*** *only one option can be selected.* |
| Severe | - Partially hydrolyzed milk protein formulas - eHFs of milk proteins - Hydrolyzed soy formulas - Hydrolyzed rice-protein formulas - Elemental formulas (based on free amino acids) - Other   ***Note:*** *only one option can be selected.* |

8. What are the 3 MAIN characteristics that you take into account when selecting a formula?

□	Type of hydrolyzed protein: __________

□	Degree of hydrolyzation: __________

□	With no lactose: __________

□	Containing pre/probiotics: __________

□	Organoleptic characteristics (smell, taste, etc.) : __________

□	Published scientific evidence: __________

□	Its proven hypoallergenicity: __________

□	Another characteristic. Please, specify: __________

***Note:*** *You can select up to a maximum of 3 options. Of those you select, write a 1, 2 or 3 on the line to indicate the ranking of preference (from most to least important, in your opinion)*

9. What is the MAIN reason you consider for prescribing an EHF that is supplemented with human milk oligosaccharides (HMOs) in a pediatric patient with CMPA?

- It promotes the normal growth of children
- It reduces the risk of infections
- It promotes the growth of beneficial bacteria and, thus, the development of the immune system
- It reduces the need for medication (such as antipyretics and antibiotics)
- Other reason. Please, specify: __________

***Note:*** *only one option can be selected.*

10. What is the MAIN reason you consider for prescribing an HMO supplemented eHF that contains purified lactose in a pediatric patient with CMPA?

- The lactose is a fundamental component in the human milk
- The lactose improves the palatability of the formula and reduces the risk of rejection
- The lactose facilitates the absorption of calcium
- The lactose acts as a prebiotic
- The lactose has a protective effect on the integrity of the intestinal mucosa
- The lactose improves the immune response and reduces the risk of infections
- Other reason. Please, specify: __________

***Note:*** *only one option can be selected.*

11. In the case of not using an eHF for the first line treatment of CMPA, what is the MAIN reason that you consider for NOT prescribing it?

- Previous anaphylaxis
- Suspected multiple food allergy
- Avoiding any potential allergenic adverse event
- Other reason. Please, specify: __________

***Note:*** *only one option can be selected*

12. What is the MAIN reason you consider for prescribing an HMO supplemented eHF that does NOT contain purified lactose?

- The presence of lactose does not produce any benefit
- Avoiding any potential secondary lactose intolerance
- Presence of CMPA-induced enteropathy
- High severity of CMPA symptoms
- Suspected multiple food allergy
- Other reason. Please, specify: __________

***Note:*** *only one option can be selected.*

**BLOCK 3. PATIENTS WITH CMPA**

To complete the following questions, please take into account the **pediatric patients that you have attended in your consultation IN THE LAST 6 MONTHS**. You must ensure that the data provided are as close as possible to what happened in your consultation.

13. How many pediatric patients have visited your consultation in the last 6 months?**:** _______
14. How many of your pediatric patients were diagnosed of **CMPA**?
15. How many of your pediatric patients with CMPA did you prescribe an **eHF**? _______
16. How many of your pediatric patients with CMPA did you prescribe an eHF that was **supplemented with HMOs**?
17. How many of your pediatric patients with CMPA did you prescribe an eHF that was **supplemented with HMOs and contained purified lactose**?

**BLOCK 4. HMOs AND LACTOSE IN THE NUTRITIONAL TREATMENT OF CMPA**

To complete the following questions, please take into account the aggregate data of the **last 5 pediatric patients with CMPA to whom you prescribed an eHF that contained purified lactose and was supplemented with HMOs**. We are not requesting individual information, but the general trend that, based on your experience with these patients as a group, may be representative of your routine clinical practice. You must ensure that the data provided are as close as possible to what happened in your consultation.

18. How many of your last 5 patients with CMPA to whom you prescribed an eHF with lactose and HMOs were in each of the following age ranges?

- 0-6 months: ______
- 7-12 months: ______
- 13-24 months: ______
- More than 24 months: ______

19. How many of your last 5 patients with CMPA to whom you prescribed an eHF with lactose and HMOs were male or female?

- Male: ______
- Female: ______

20. How many of your last 5 patients with CMPA to whom you prescribed an eHF with lactose and HMOs showed the following types of allergy?

- IgE-mediated CMPA: ______
- Non-IgE-mediated CMPA (cellular): ______
- Mixed mechanisms (IgE/cellular): ______

21. How many of your last 5 patients with CMPA to whom you prescribed an eHF that contained purified lactose and was supplemented with HMOs showed the following severity of symptoms:

- Mild: ______
- Moderate: ______
- Severe: ______

22. How many of your last 5 patients with CMPA to whom you prescribed an eHF with lactose and HMOs had the following symptoms?

- Cutaneous: ______
- Digestives: ______
- Respiratory: ______
- Crying and/or irritability: ______
- Rejection/difficulty for eating: ______
- Anaphylaxis: ______

23. How many of your last 5 patients with CMPA to whom you prescribed an eHF with lactose and HMOs showed an IMPROVEMENT in each one of the following symptoms?

- Cutaneous: ______
- Digestives: ______
- Respiratory: ______
- Crying and/or irritability: ______
- Rejection/difficulty for eating: ______
- Anaphylaxis: ______
- Reduce the number of stools: ______
- Achieve a normal stool consistency: ______
- The gastroesophageal reflux: ______

24. How long did it take for clinical symptoms to disappear after starting the treatment with the eHF with lactose and HMOs?

- Hours-1 day
- 2-3 days
- 4-6 days
- 7-14 days
- 5-21 days
- >28 days

***Note:*** *only one option can be selected.*

25. Regarding growth and development (considering the standard growth charts, for instance those from the World Health Organization), in your experience…?

- The use of an eHF with lactose and HMOs provides AS OPTIMAL growth and development AS with other eHF (without HMOs and lactose)
- The use of an eHF with lactose and HMOs provides LESS OPTIMAL growth and development than other eHF (without HMOs and lactose)
- I have no data to evaluate it

***Note:*** *only one option can be selected.*

26. Regarding lower/upper respiratory tract infections, in your experience…?

- The incidence of respiratory infections is observed to be THE SAME when using an eHF with lactose and HMOs than other eHF (without lactose and HMOs)
- The incidence of infections is observed to be HIGHER with an eHF with lactose and HMOs than other eHF (without lactose and HMOs)
- The incidence of infections is observed to be LOWER with an eHF with lactose and HMOs than other eHF (without lactose and HMOs)
- I have no data to evaluate it

***Note:*** *only one option can be selected.*

27. Regarding the use of medications (such as antipyretics and antibiotics), in your experience…?

- The use of medications is THE SAME when using an eHF with lactose and HMOs than other eHF (without lactose and HMOs)
- The use of medications is HIGHER with an eHF with lactose and HMOs than other eHF (without lactose and HMOs)
- The use of medications is LOWER with an eHF with lactose and HMOs than other eHF (without lactose and HMOs)
- I have no data to evaluate it

***Note:*** *only one option can be selected.*

28. Regarding the patient’s visits to your consultation, in your experience…?

- The number of visits is THE SAME when using an eHF with lactose and HMOs than other eHF (without lactose and HMOs)
- The number of visits is HIGHER with an eHF with lactose and HMOs than other eHF (without lactose and HMOs)
- The number of visits is LOWER with an eHF with lactose and HMOs than other eHF (without lactose and HMOs)
- I have no data to evaluate it

***Note:*** *only one option can be selected.*

29. In your experience, what are the 3 MAIN characteristics for choosing an eHF with lactose and HMOs for the treatment of CMPA?

□	It is inspired by breast milk: __________

□	It is effective for the relief of symptoms: __________

□	It promotes the maturation of the immune system: __________

□	The preferred flavor: __________

□	Another characteristic. Please, specify: __________

***Note:*** *You can select up to a maximum of 3 options. Of those you select, write a 1, 2 or 3 on the line to indicate the ranking of preference (from most to least important, in your opinion)*

30. In your experience, what is your satisfaction on the use of eHFs with lactose and HMOs?

- Very satisfied
- Satisfied
- Neither satisfied nor dissatisfied
- Dissatisfied
- Very dissatisfied

***Note:*** *only one option can be selected.*

31. Regarding the efficacy of eHFs with lactose and HMOs for the treatment of CMPA, you think that their use is…:

- …very effective
- …effective but not as expected
- …not effective
- I have no data to evaluate it

***Note:*** *only one option can be selected.*

# **APPENDIX S2**

**Supplementary Table 1**. Autonomous communities where the participating pediatricians attended infants with CMPA

|  | **Valor** |
| --- | --- |
| Comunidad de Madrid | 61 (31.9) |
| Cataluña | 53 (27.7) |
| Valencia | 39 (20.4) |
| Andalucía | 22 (11.5) |
| País Vasco | 4 (2.1) |
| Castilla La Mancha | 3 (1.6) |
| Murcia | 3 (1.6) |
| Aragón | 2 (1.0) |
| Asturias | 1 (0.5) |
| Baleares | 1 (0.5) |
| Castilla León | 1 (0.5) |
| Navarra | 1 (0.5) |

CMPA, cow’s milk protein allergy

# **APPENDIX S3**

Supplementary Table 2. Diagnosis of CMPA and evolution of symptoms in infants

|  | **n (%)** |
| --- | --- |
| **Main base for the diagnosis of CMPA in infants** |  |
| Symptomatology (compatible with CMPA) | 152 (79.6) |
| Elimination diet | 18 (9.4) |
| Oral food challenge test | 15 (7.9) |
| Skin prick test | 4 (2.1) |
| Serum specific IgE test | 2 (1.0) |
| **Participants using any tool for the detection and evolution of potential CMPA symptoms** | 87 (45.5) |

CMPA, cow’s milk protein allergy

# **APPENDIX S4**

**Supplementary Figure 1**. Main characteristics taken into account when selecting a formula for infants with CMPA

CMPA, cow’s milk protein allergy

# **APPENDIX S5**

**Supplementary Figure 2**. Main reasons for prescribing an eHF supplemented with HMO in infants with CMPA

CMPA, cow’s milk protein allergy

# **APPENDIX S6**

**Supplementary Figure 3**. Reasons for prescribing an eHF that contains purified lactose in infants with CMPA

eHF, extensively hydrolyzed formula; CMPA, cow’s milk protein allergy

**APPENDIX S7**

Supplementary Table 3. Characteristics of the last five infants who received an eHF supplemented with purified lactose and HMOs

|  | **% Mean** | **% Standard deviation** |
| --- | --- | --- |
| **Sex** |  |  |
| Male | 55.8 | 17.5 |
| Female | 44.2 | 17.5 |
| **Range of age, months** |  |  |
| 0-6 | 81.8 | 21.5 |
| 7-12 | 15.9 | 19.1 |
| 13-24 | 1.7 | 5.6 |
| >24 | 0.6 | 3.5 |
| **Type of allergy** |  |  |
| Non-IgE-mediated CMPA (cellular) | 75.5 | 27.9 |
| IgE-mediated CMPA | 20.6 | 25.1 |
| Mixed mechanisms (IgE/cellular) | 3.9 | 9.6 |
| **Severity of symptoms** |  |  |
| Mild | 62.2 | 27.4 |
| Moderate | 33.5 | 25.5 |
| Severe | 4.3 | 10.3 |

eHF, extensively hydrolyzed formula; HMOs, human milk oligosaccharides; CMPA, cow’s milk protein allergy

# **APPENDIX S8**

**Supplementary Figure 4**. Main characteristics for choosing an eHF with purified lactose and HMOs

eHF, extensively hydrolyzed formula; HMOs, human milk oligosaccharides

# **APPENDIX S9**

**Supplementary Figure 5**. Satisfaction with an eHF with purified lactose and HMOs

eHF, extensively hydrolyzed formula; HMOs, human milk oligosaccharides
